# Extracellular Matrix Tissue Patch for Aortic Arch Repair in Pediatric Cardiac Surgery: A Single-Center Experience

**DOI:** 10.3390/jcm14113955

**Published:** 2025-06-03

**Authors:** Marcin Gładki, Anita Węclewska, Paweł R. Bednarek, Tomasz Urbanowicz, Anna Olasińska-Wiśniewska, Bartłomiej Kociński, Marek Jemielity

**Affiliations:** 1 Department of Pediatric Cardiac Surgery, Poznan University of Medical Sciences, 60-572 Poznań, Poland; 2 Department of Cardiac Surgery and Transplantology, Poznan University of Medical Sciences, 61-848 Poznań, Poland

**Keywords:** pediatric cardiac surgery, congenital heart surgery, congenital heart disease, aortic arch repair, extracellular matrix patch

## Abstract

**Introduction**: Among aortic diseases in children, congenital defects such as coarctation of the aorta (CoA), interrupted aortic arch (IAA), hypoplastic aortic arch (HAA), and hypoplastic left heart syndrome (HLHS) predominate. Tissue patches are applied in pediatric cardiovascular surgery for the repair of congenital aortic defects as a filling material to replenish missing tissue or as a substitute material for the complete reconstruction of the vascular wall along the course of the vessel. This retrospective single-center study aimed to present the safety and feasibility of extracellular matrix (ECM) biological scaffolds in pediatric aortic surgery. **Patients and methods**: There were 26 patients (17 newborns and nine children), who underwent surgical procedures in the Department of Pediatric Cardiac Surgery (Poznań, Poland) between 2023 and 2024. The patients’ population was divided into two subgroups according to the hemodynamic nature of the primary diagnosis of the congenital heart defect and the performed pediatric cardiovascular surgery. The first group included 18 (72%) patients after aortic arch repair for interrupted aortic arch and/or hypoplastic aortic arch, while the second group included seven (28%) patients after aortopulmonary anastomosis. In the first group, patches were used to reconstruct the aortic arch by forming an artificial arch with three separate patches sewn together, primarily addressing the hypoplastic or interrupted segments. In the second group, patches were applied to augment the anastomosis site between the pulmonary trunk and the aortic arch, specifically at the connection points in procedures, such as the Damus–Kaye–Stansel or Norwood procedures. The analysis was based on data acquired from the national cardiac surgery registry. **Results**: The overall mortality in the presented group was 15%. All procedures were performed using median sternotomy with a cardiopulmonary bypass. The cardiopulmonary bypass (CPB) and aortic cross-clamp (AoX) median times were 144 (107–176) and 53 (33–79) min, respectively. There were two (8%) cases performed in deep hypothermic circulatory arrest (DHCA). The median postoperative stay in the intensive care unit (ICU) was 284 (208–542) h. The median mechanical ventilation time was 226 (103–344) h, including 31% requiring prolonged mechanical ventilation support. Postoperative acute kidney failure requiring hemodiafiltration (HDF) was noticed in 12% of cases. Follow-up data, collected via routine transthoracic echocardiography (TTE) and clinical assessments over a median of 418 (242.3–596.3) days, showed no evidence of patch-related complications such as restenosis, aneurysmal dilation, or calcification in surviving patients. One patient required reintervention on the same day due to a significantly narrow ascending aorta, unrelated to patch failure. No histological data from explanted patches were available, as no patches were removed during the study period. The median (Q1–Q3) hospitalization time was 21 (16–43) days. **Conclusions**: ProxiCor^®^ biological patches derived from the extracellular matrix can be safely used in pediatric patients with congenital aortic arch disease. Long-term follow-up is necessary to confirm the durability and growth potential of these patches, particularly regarding their resistance to calcification and dilation.

## 1. Introduction

Congenital heart defects are present in approximately 1% of births in developed countries. Among aortic diseases in children, congenital defects such as coarctation of the aorta (CoA), interrupted aortic arch (IAA), hypoplastic aortic arch (HAA), and hypoplastic left heart syndrome (HLHS) predominate. For most of these defects, complete correction is possible (for hypoplastic aortic arch, coarctation of the aorta, and interrupted aortic arch), which involves restoring anatomy to normal. Children can lead relatively normal lives in the future. In some patients, aortic defects are also accompanied by intracardiac defects that are not correctable. The most common is hypoplastic left heart syndrome, requiring conversion to a univentricular heart as a palliative treatment. This pathway has a number of complications and is associated with an unequivocally worse prognosis [1]. Moreover, some children require elective reoperation as a part of multistage surgical treatment related to their growth or as a consequence of replaced material degeneration.

Tissue patches are applied in pediatric cardiovascular surgery for the repair of the congenital aortic defects as a material to replenish missing tissue or as a material for the complete reconstruction of the vascular wall along the course of the vessel. In pediatric cardiac surgery, biological tissues, such as homografts or autologous and animal pericardium, or synthetic materials are used [2,3]. Contrary to cardiac surgery in adult patients, the implantation of prefabricated synthetic conduits for aortic substitution is not an option in pediatric cardiac surgery [4].

Despite their common use in pediatric cardiovascular surgery for managing congenital heart defects, there are several issues related to the above-mentioned materials. Biocompatible synthetic polymers are associated with a risk of stenosis, thromboembolization, and infection [3] and most importantly, in the pediatric population, a lack of growth potential. Biological tissues, in turn, such as autologous and bovine pericardium, may be affected by calcification and shrinkage over time [2,5]. The necessity to overcome these disadvantages has prompted the development of novel tissue engineering techniques focused on decellularized biological scaffolds. They are characterized by biocompatibility, the lack of scar tissue formation, and potential for constructive remodeling via the organism’s cells’ ability to repopulate the material [2].

Several studies and reviews [6,7] have shown the feasibility and disadvantages of material used in pediatric cardiovascular surgery. However, data concerning the use of ProxiCor^®^ (Elutia, Inc., formerly Aziyo Biologics, Inc., Roswell, NM, USA), an extracellular matrix (ECM) derived from porcine small intestinal submucosa (SIS), in children, particularly those with aortic arch defects, are still lacking.

This retrospective single-center study aimed to present the safety and feasibility of acellular extracellular matrix biological scaffolds in pediatric aortic surgery based on data acquired from the national cardiac surgery registry (pol. *Krajowy Rejestr Operacji Kardiochirurgicznych*—KROK, Children’s Memorial Health Institute, Warsaw, Poland; https://krok.csioz.gov.pl/krok/).

## 2. Patients and Methods

### 2.1. Patients

There were 26 patients (17 (65%) males and 9 (35%) females), including 17 newborns with a median age of 1 (1–3.5 days) and 9 children with a median age of 367 (179–3600) days, who underwent surgical procedures on the thoracic aorta at the Department of Pediatric Cardiac Surgery (Poznań, Poland) between 2023 and 2024 following the introduction of ProxiCor^®^ for Cardiac Tissue Repair (CTR) (Elutia, Inc., formerly Aziyo Biologics, Inc., Roswell, NM, USA) into the portfolio of available implantable patches in local cardiac surgery operating theatre.

The patients’ population was divided into 2 subgroups according to the hemodynamic nature of the primary diagnosis of congenital heart defect and the cardiovascular surgery operation performed. The first group included 18 patients after aortic arch repair for interrupted aortic arch and/or hypoplastic aortic arch, including complete reconstruction of aortic arch using 3 separate patches sewn together forming artificial aortic arch (Supplementary Video S1), while the second group included 7 patients after aortopulmonary anastomosis (of the pulmonary trunk with the aortic arch) for management of univentricular heart (including 3 patients after the Damus–Kaye–Stansel procedure) and 4 patients with hypoplastic left heart syndrome after the Norwood procedure.

In the first group, the ProxiCor^®^ patches were primarily used to reconstruct the hypoplastic or interrupted segments of the aortic arch, ensuring continuity and adequate diameter of the vessel. In cases of interrupted aortic arch, patches were sutured to bridge the gap between the proximal and distal segments. For hypoplastic aortic arch, patches were used to augment the narrowed segments, often extending from the ascending aorta to the descending aorta. In the second group, patches were applied at the anastomosis site between the pulmonary trunk and the aortic arch to reinforce the connection and prevent narrowing, particularly critical in high-pressure environments like the Damus–Kaye–Stansel or Norwood procedures. The subgroup division was based on the distinct hemodynamic challenges: the first group involved high-pressure aortic reconstructions, while the second group addressed complex aortopulmonary connections in univentricular physiology, allowing for tailored surgical approaches and outcome analysis.

The median (Q1–Q3) height and mass were 54 (51–59) centimeters and 3.4 (3.1–4.0) kilograms, respectively.

There were five patients who underwent elective reoperations (as a part of multi-staged surgical treatment) in the presented analysis, indicating subsequent heart surgeries during their lifetime.

The demographic and clinical characteristics are presented in Table 1.

All patients underwent routine postoperative transthoracic echocardiography (TTE) during follow-up, from the first-ever patch application in first-ever patient (2023) till the completion of the statistical analysis (2025), in median time (Q1–Q3) of 418 (242.3–596.3) days, as presented in Table 2.

All patients underwent routine postoperative transthoracic echocardiographic follow-up. Patients were recruited continuously over a two-year period, resulting in variable follow-up durations depending on the date of surgery. Accordingly, patients who were operated on earlier had up to two years of follow-up, while those treated later had only several months of observation. The overall median (Q1–Q3) follow-up time was 418 (242.3–596.3) days, as presented in Table 2.

### 2.2. Methods

ProxiCor^®^ is a ready-to-use 4-ply biological tissue patch derived from extracellular matrix, available in a rectangular configuration with various size options to accommodate surgical requirements. The patch exhibits structural rigidity, facilitating precise trimming with surgical scissors. Upon hydration, the material gains pliability and mechanical integrity, enhancing its conformability and integration with adjacent anatomical structures, fitting in shape without sharp-angled kinking. All patches underwent routine pre-implantation hydration in isotonic saline solution to restore tissue pliability and facilitate handling during surgery. This step was performed universally, regardless of subsequent modifications. In cases requiring reconstruction of particularly delicate or hypoplastic structures—most notably, in neonates with critically underdeveloped vasculature—an additional preparation step was employed, consisting of the manual delamination of one or more extracellular matrix layers. This process yielded a thinner and more compliant patch, tailored to the anatomical and mechanical demands of fragile neonatal tissues. The decision to delaminate was made intraoperatively, based on the surgeon’s assessment of tissue characteristics, with the aim of optimizing graft conformity and integration in anatomically constrained or low-pressure hemodynamic environments. No significant differences in outcomes were observed between the two preparation methods, though delamination was more commonly used in neonates due to their fragile tissues. The standard surgical technique for anastomosing the patch to the native tissues is running continuous suture.

All patients were operated on through a median sternotomy approach with cardiopulmonary bypass (CPB) application. A single venous cannula was inserted into the right atrium, while an arterial cannula was inserted into the ascending aorta. After reaching an appropriate temperature, the cannula was repositioned into the brachiocephalic trunk for selective cerebral perfusion. This approach leads to a reduction in possible adverse neurological effects [8,9]. In all patients, the heart function was routinely stopped using cold blood del Nido cardioplegic solution [10]. In 2 patients with the smallest anatomical dimensions of vessels, it was decided to apply deep hypothermic circulatory arrest (DHCA). This technique involves arterial cannulation of the ascending aorta, and then, after achieving profound hypothermia, to decannulate and perform the procedure under time-limited complete circulatory arrest, when needed.

We perform surgery within the aortic arch in children using deep intraoperative hypothermia [11]. Therapeutic hypothermia reduces the organs’ need for oxygen and nutrients, which makes it possible to perform the surgery in complete circulatory arrest when required [12]. This allows much better visualization of the surgical field during the procedure. The protocol for deep hypothermia [13] is based on maintaining a maximum gradient of 5 °C between the temperature measured at the arterial line set on the heat exchanger and the highest body temperature measured from either the esophageal or rectal probe.

### 2.3. Statistical Analysis

The Shapiro–Wilk test was used to evaluate the data distribution. Since data did not confirm normal distribution, they were expressed as the median and 25–75th percentile (Q1–Q3). Categorical variables were presented as numbers and percentages. Statistical analysis was performed using JASP software (JASP Team; 2020; version 0.13.1).

The consent of individual patients was waived due to the retrospective nature of the study, as determined by the institutional bioethics committee board.

## 3. Results

The median (Q1–Q3) age, body mass, and height of the enrolled population were 12 (8–51) days, 3.4 (3.1–4.0) kg, and 54 (51–59) cm, respectively. In three (12%) cases, the primary procedure needed to be redone. The reasons for reoperation included the following: one case of a significantly narrow ascending aorta detected on postoperative echocardiography requiring same-day reintervention, one case of the suboptimal alignment of anatomical structures causing hemodynamic instability, and one case of residual stenosis at the anastomosis site, all addressed within the first week post-surgery. These reoperations were not directly attributable to patch failure but rather to anatomical or procedural challenges.

Only one (4%) of the patients after aortoplasty of an extreme hypoplastic aortic arch required a reintervention to expand the anatomical scope on the same day of the primary operation. A significantly narrow ascending aorta was revealed via a postoperative echocardiography. In the follow-up period to date (2025), no patients have been observed to develop restenosis requiring further reoperation.

The early and overall mortality in the presented group was four (15%) patients. The causes of death were postoperative multiorgan failure in three (12%) patients and cerebral edema despite decompressive craniectomy in one (4%) case, none of which were directly linked to the ProxiCor^®^ patch performance. The 15% mortality rate aligns with reported rates for complex congenital heart defect repairs in neonates, which range from 10 to 20% in high-volume centers [1], though direct comparisons are limited by variations in patient complexity and surgical techniques. The median (Q1–Q3) hospitalization time was 21 (16–43) days, as presented in Table 3.

All procedures were performed using a median sternotomy with a cardiopulmonary bypass. The median (Q1–Q3) CPB and aortic cross-clamp (AoX) times were 144 (107–176) min and 53 (33–79) min, respectively. There were two (8%) cases performed in DHCA with a time of 22 and 27 min, respectively.

The mean lowest body core temperature during the procedures was 16.6 °C, measured rectally. The DHCA was applied in two (8%) cases. The indications for the technique were to obtain the optimal operating conditions for strategic structures in case of complex anatomical conditions in vivo due to the critically underdeveloped vessels.

For postoperative low cardiac output syndrome, diagnosed in three (12%) patients, veno-arterial extracorporeal oxygenation (ECMO) therapy was deemed mandatory.

The median (Q1–Q3) postoperative stay in the intensive care unit (ICU) was 284 (208–542) h. The median (Q1–Q3) mechanical ventilation time was 226 (103–344) h, including eight (31%) requiring prolonged mechanical ventilation support (over 7 days). Postoperative acute kidney failure requiring continuous renal replacement therapy (CRRT) was noticed in three (12%) cases, including continuous veno-venous hemodiafiltration (CVVHDF) in one (4%) patient and two (8%) patients during veno-arterial ECMO, depending on the connection configuration of the dialyzer system to the patient’s circulatory system or ECMO circuit. The permanent pacemaker was implanted in one (4%) patient due to a complete atrioventricular (AV) block as presented in Table 4.

## 4. Discussion

Our study demonstrates the feasibility and safety of using acellular extracellular matrix biological scaffolds in pediatric aortic surgery. The results suggest that this biological material may serve as a viable option, particularly in neonates and infants with complex aortic arch defects.

Compared with current techniques, the past surgical approach to congenital aortic arch defects historically relied on autologous pericardium [14] or synthetic materials such as expanded polytetrafluoroethylene (Gore-Tex^®^, W.L. Gore & Associates, Inc., Newark, DE, USA) and polyethylene terephthalate (Dacron^®^, DuPont Corporation, Wilmington, DE, USA) [15]. However, their use has become highly limited due to their rigidity and the associated risk of sharp-angled kinking, which could lead to hemodynamic complications. Additionally, these materials lack growth potential and have been associated with restenosis and calcification, making them suboptimal for use in neonates and infants [16]. In contrast, patches made from autologous pericardium or synthetic materials may be too thin to withstand the high-pressure flow in the aorta, potentially leading to aneurysmal dilation [17,18] or structural failure over time. For example, synthetic materials like Gore-Tex^®^ typically require replacement within 5–10 years due to calcification or stenosis, while autologous pericardium may calcify within 3–7 years, depending on patient age and hemodynamic stress. ProxiCor^®^ patches, in our study, showed no signs of calcification or dilation over a median follow-up of 418 days, though longer-term data are needed to confirm their durability. This limitation further underscores the need for alternative materials that provide both durability and the ability to integrate with native tissue. Our findings indicate that acellular extracellular matrix biological scaffolds may overcome some of these challenges, as evidenced by the lack of restenosis requiring reoperation in the follow-up period.

Several studies have reported differential results for biological cardiovascular patches used in pediatric cardiovascular surgery for the repair of congenital heart defects. Weis et al. [2] presented 57 children treated surgically due to atrial and ventricular defects with the use of biological scaffold materials obtained from porcine small intestinal submucosa (CorMatrix^®^ (CM); CorMatrix^®^ Cardiovascular, Inc., Roswell, GA, USA). Since the development of stenosis or aneurysms was observed, they concluded that CorMatrix is safe, with good postoperative results, but associated with a higher reintervention rate, compared with autologous pericardium use. Similarly, van Beynum et al. [19] in an analysis of 36 patients treated with aortic arch repair with the use of homograft or tissue-engineered bovine pericardium (CardioCel^®^, Admedus Regen Pty, Ltd., Toowong, Australia) reported a higher rate of intervention for restenosis in the CardioCel^®^ group. In contrast, Witt et al. [20] showed good results for porcine small intestinal submucosa patches in 37 patients with different cardiovascular reconstructions, with a high patency of the material and a low risk of restenosis, especially in patches that cover the majority of the vessel circumference. Neethling et al. [21] underlined the excellent long-term results of tissue-engineered ADAPT^®^ bovine pericardial patches (ABPP; CardioCel^®^, Celxcel Pty Ltd., Perth, Western Australia) without signs of calcification or graft-related morbidity and mortality.

The performance of extracellular matrix scaffolds may depend on the implantation site [22], which is associated with the working environment and mechanical loading. Beneficial effects were reported in atrial and ventricular septal defect closures, while at high-pressure sites such as the aortic valve, the results showed the need for reintervention [23]. Notably, no calcifications were detected during imaging examinations or reoperations [23].

In our study population, we performed surgeries with the use of ProxiCor^®^, a decellularized non-crosslinked extracellular matrix biological scaffold primarily designed for the repair of the pericardium. The beneficial effects were presented in a large prospective multi-center RECON study [24]. By decellularization, most of the tissue’s cellular and antigenic components are removed, leaving mechanical properties similar to human tissue [25,26]. The material is naturally derived, which provides several advantages over synthetic ones. It may serve as a substitute for damaged tissue due to having a high content of natural components, including collagens, fibronectin, elastin, glycosaminoglycans, and growth factors [27]. In vivo, the extracellular matrix is repopulated by the patient’s own cells enabling rapid tissue repair. The small intestinal submucosa extracellular matrix was proven to demonstrate antimicrobial activity and reduced inflammatory response during remodeling and tissue healing, further supporting angiogenic processes [28,29]. Therefore, the small intestinal submucosa extracellular matrix carries a lower risk of reactive inflammation, calcification, and local material stiffness [27].

In our experience, ProxiCor^®^ patches were implanted successfully in all patients, without material-related postoperative morbidity or mortality. The observed low rate of early reintervention (one case on the same day as the primary operation) in our study suggests that ProxiCor^®^ biological scaffolds provide adequate mechanical support while potentially facilitating native tissue remodeling to histologically identical and functional, site-specific aortic tissue. This aligns with findings from previous studies on extracellular-matrix-derived materials in pediatric cardiovascular surgery, which suggest their ability to integrate with host tissue and support neovascularization, allowing capillary in-growth and infiltration during the angiogenic process.

Follow-up assessments, conducted via routine transthoracic echocardiography (TTE) and clinical evaluations, revealed no patch-related complications such as restenosis, dilation, or calcification over a median of 418 days. However, explanted patches were absent during the study period, which precluded the histological analysis, which would have provided insights into tissue remodeling and long-term integration. Compared to other materials, such as CardioCel^®^, which has shown restenosis rates of up to 20% within 2 years in some studies, our findings suggest ProxiCor^®^ may offer comparable or improved early outcomes, though an extended follow-up is needed to assess long-term performance.

Despite the promising results, the study population faced significant perioperative risks. The observed perioperative mortality rate reflects the severity of the underlying congenital heart defects and the complexity of the surgical procedures rather than a direct consequence of biological scaffold use. Three deaths were associated with the failure of veno-arterial ECMO therapy, while one resulted from complicated cerebral edema despite a decompressive craniectomy being performed. These complications highlight the need for refined perioperative management strategies in this challenging high-risk group.

The requirement for DHCA emphasizes the complexity of aortic arch reconstruction in neonates. While DHCA provides optimal surgical exposure and minimizes ischemic injury to the heart, it carries potential risks, including neurological complications. Our results indicate that adherence to a strict intraoperative hypothermia protocol, with a maximum temperature gradient of 5 °C, contributes to minimizing adverse neurological outcomes.

The successful use of acellular extracellular matrix biological scaffolds in our study supports their potential role as a preferred patch material in pediatric aortic surgery. Their biocompatibility, potential for remodeling, and avoidance of synthetic materials make them particularly attractive for clinical practice in neonates and infants whose long-term vascular growth potential could be improved. However, further follow-up is needed to assess whether these materials provide durable outcomes and reduce the need for reintervention in later life stages.

### Study Limitations

This was a retrospective, single-center analysis with a relatively small sample size. Additionally, while short-term outcomes are promising, long-term follow-up is essential to determine the durability and growth potential of acellular extracellular matrix biological scaffolds. The lack of histological data from explanted patches limits insights into tissue remodeling, and the absence of a direct comparison group (e.g., patients treated with synthetic patches) restricts our ability to definitively assess ProxiCor^®^’s superiority. The 15% mortality rate, while consistent with complex neonatal repairs, cannot be fully contextualized without multi-center data on similar patient cohorts. Future multicenter studies with larger cohorts and extended follow-up periods are warranted to validate our findings and establish standardized guidelines for, as well as the applicability and limitations of, their use in pediatric cardiovascular surgery for the surgical treatment of congenital heart defects, especially aortic defects.

## 5. Conclusions

Acellular extracellular matrix biological scaffolds represent a promising alternative for aortic arch reconstruction in neonates and infants with congenital heart defects. Their biocompatibility and potential for integration with host tissues suggest advantages over traditional synthetic materials. While our findings demonstrate favorable early outcomes, further research is needed to confirm their long-term efficacy and safety.

## Figures and Tables

**Table 1:** Demographic and clinical characteristics.

Demography	Parameters	Analyzed Group (*n* = 26)
Sex	males (%)/females (%)	17 (65)/9 (35)
Age	1. Newborns	17 (65)
	Median age (days) (Q1–Q3)	1 (1–3.5)
	2. Children	9 (35)
	Median age (days) (Q1–Q3)	367 (179–3600)
Anthropometry	3. Median height (cm) (Q1–Q3)	54 (51–59)
	4. Median height (kg) (Q1–Q3)	3.4 (3.1–4.0)
Primary diagnosis	Interrupted aortic arch (IAA) (*n*) (%)	5 (19)
	Hypoplastic aortic arch (HAA) (*n*) (%)	9 (35)
	IAA + HAA (*n*) (%)	2 (8)
	Aortic stenosis (AS) (*n*) (%)	2 (8)
	Univentricular heart (*n*) (%)	8 (31)
Previous surgeries	Interrupted aortic arch (IAA) (*n*)	1
	Arterial switch operation (ASO) * with IAA (*n*)	1
	Left ventricular outflow tract obstruction (LVOTO) (*n*)	1
	Hypoplastic aortic arch (HAA) (*n*)	2

* for transposition of the great arteries (TGA). Abbreviations: IAA—interrupted aortic arch, HAA—hypoplastic aortic arch, ASO—arterial switch operation (Jatene procedure).

**Table 2:** Descriptive statistics.

Parameters	Time
Median (Q1–Q3) (days)	418
25th percentile (days)	242.3
50th percentile (days)	418
75th percentile (days)	596.3

**Table 3:** Hospitalization data.

Parameters		Analyzed Group (*n* = 26)
Mortality	Overall (*n*) (%)	4 (15)
	Early (*n*) (%)	3 (11)
	Late (*n*) (%)	1 (3)
Hospitalization	Overall (days) (median) (Q1–Q3)	21 (16–43)
	ICU (hours) (median) (Q1–Q3)	284 (208–543)

Abbreviations: ICU—intensive care unit.

**Table 4:** Intra- and postoperative characteristics.

Parameters		Analyzed Group (*n* = 26)
Intraoperative characteristics	CPB time (minutes) (median) (Q1–Q3)	144 (107–176)
	AoX time (minutes) (median) (Q1–Q3)	53 (33–79)
	DHCA (*n*) (%)	2 (8%)
	DHCA time (minutes)	22, 27
Postoperative characteristics	Mechanical ventilation (minutes) (median) (Q1–Q3)	226 (103–344)
	Prolonged ventilation * (*n*) (%)	8 (31)
	Continuous renal replacement therapy (CRRT) (*n*) (%)	3 (12)
	PPM implantation (*n*) (%)	1 (4)

* above 7 days. Abbreviations: AoX—aortic cross-clamp, CPB—cardiopulmonary bypass, CRRT—continuous renal replacement therapy, DHCA—deep hypothermic circulatory arrest, PPM—permanent pacemaker.

## Data Availability

The raw data supporting the conclusions of this article will be made available by the authors on request.

## Supplementary Materials

The following supporting information can be downloaded at: https://www.mdpi.com/article/10.3390/jcm14113955/s1, Video S1: Aortic arch repair.

## Abbreviations

The following abbreviations are used in this manuscript:

ABPP	ADAPT^®^ bovine pericardial patch
ASO	Arterial switch operation
AV	Atrioventricular
CM	CorMatrix^®^
CoA	Coarctation of the aorta
CPB	Cardiopulmonary bypass
CRRT	Continuous renal replacement therapy
DHCA	Deep hypothermic circulatory arrest
DKS	Damus–Kaye–Stansel procedure
ECM	Extracellular matrix
ECMO	Extracorporeal membrane oxygenation
HAA	Hypoplastic aortic arch
HDF	Hemodiafiltration
HLHS	Hypoplastic left heart syndrome
IAA	Interrupted aortic arch
ICU	Intensive care unit
KROK	*Krajowy Rejestr Operacji Kardiochirurgicznych*—national cardiac surgery registry
LVOTO	Left ventricular outflow tract obstruction
SIS	Small intestinal submucosa
TTE	Transthoracic echocardiography
